# Hemodialysis as a Risk Factor for Lower Right Internal Jugular Stenosis in Cardiac Surgery Patients: A Retrospective Single-Center Study

**DOI:** 10.3390/jcm10051042

**Published:** 2021-03-03

**Authors:** Jae-Woo Ju, Yoomin Oh, Hyo Jun Yang, Seohee Lee, Jinyoung Bae, Karam Nam, Youn Joung Cho, Yunseok Jeon, Tae Kyong Kim

**Affiliations:** 1 Department of Anesthesiology and Pain Medicine, Seoul National University Hospital, Seoul National University College of Medicine, Seoul 03080, Korea; jujw701@gmail.com (J.-W.J.); yoominoh@gmail.com (Y.O.); hyojun35@naver.com (H.J.Y.); leesen34@gmail.com (S.L.); baejy88@gmail.com (J.B.); karamnam@gmail.com (K.N.); mingming7@gmail.com (Y.J.C.); jeonyunseok@gmail.com (Y.J.); 2 Department of Anesthesiology and Pain Medicine, Seoul Metropolitan Government-Seoul National University Boramae Medical Center, Seoul National University College of Medicine, Seoul 07061, Korea

**Keywords:** cardiac surgery, right internal jugular stenosis, hemodialysis, central venous catheterization, computed tomography

## Abstract

Lower right internal jugular vein (RIJ) stenosis has been reported as a common cause of RIJ catheterization failure. However, the risk factors for lower RIJ stenosis in patients undergoing cardiac surgery is unclear. We reviewed the electronic medical records of all adult patients who had undergone cardiac operations in a single tertiary university hospital from January 2014 to January 2016. Patients were excluded if they were lack of preoperative contrast-enhanced chest computed tomography (CT) studies. Lower RIJ stenosis was defined as a ratio of cross-sectional area at the smallest level to cross-sectional area at the largest level less than 25%. Multivariable logistic regression analyses were used to investigate the risk factors for lower RIJ stenosis. A sensitivity analysis was also conducted using a cross-sectional area ratio of under 20%. The analysis included 889 patients, and the incidence of lower RIJ stenosis was 3.9%. The multivariable logistic regression analyses revealed that hemodialysis was an independent risk factor for lower RIJ stenosis (OR, 3.54; 95% CI, 1.472–8.514). Sensitivity analysis provided that hemodialysis (OR, 10.842; 95% CI, 3.589–32.75) was a significant predictor of cross-sectional area ratio <20%. Preoperative hemodialysis are significantly associated with an increased risk of lower RIJ stenosis in patients undergoing cardiac surgery. Extra care is needed during central venous catheterization in hemodialysis patients undergoing cardiac surgery.

## 1. Introduction

In cardiac surgery, central venous catheterization (CVC) is mandatory due to the high incidence of blood transfusion, vasoactive drug infusions, and the need for pulmonary artery pressure monitoring. Moreover, as minimally invasive cardiac surgery becomes more popular, cannulation into central veins such as the internal jugular or femoral vein is also becoming more frequent. Right internal jugular vein (RIJ) catheterization is favored over subclavian vein or femoral vein catheterization in cardiac surgery patients because it provides the shortest and straightest path to the heart [1], has a favorable distance from the surgical field [2], and makes it easy to insert a pulmonary artery catheter [3]. Moreover, the incidence of catheter-related complications, such as pneumothorax, catheter malposition, and thoracic duct injury, is lower in RIJ catheterization compared with other sites [4,5]. However, it is sometimes necessary to place a central venous catheter on the left internal jugular vein (LIJ) or other central veins due to the unexpected failure of RIJ catheterization in cardiac surgery patients [6].

Lower RIJ stenosis has been reported to be a cause of RIJ catheterization failure [7]. Although the introduction of ultrasound guidance techniques has increased the success rate of internal jugular vein (IJV) catheterization [6], the use of ultrasonography alone cannot guarantee the patency of the intrathoracic pathway for central venous catheters. Previous studies have reported that RIJ stenosis is common in hemodialysis patients, with an incidence of 0% to 7.7% [8,9,10]. These patients may have catheter dysfunction or develop symptoms such as arm edema, ulceration, swelling, venous dilation, or superior vena cava syndrome [11,12]. Previous studies have shown that the risk factors for RIJ stenosis include the number of hemodialysis sessions and the number and duration of previous indwelling central vein catheters [11,13,14].

Cardiac surgery patients have many risk factors that can potentially develop RIJ stenosis. Risk factors for venous thromboembolism, such as hypertension, dyslipidemia, atherosclerosis, and heart failure [15,16,17], overlap with risk factors for coronary artery disease or valve disease [18]. There are also many elderly patients on hemodialysis who require cardiac surgery. However, the actual incidence of RIJ stenosis and risk factors for RIJ stenosis in cardiac surgery patients have not been investigated. The aim of the present study was to retrospectively investigate the incidence of and risk factors for lower RIJ stenosis in patients undergoing cardiac surgery.

## 2. Materials and Methods

### 2.1. Ethics Committee Approval

Ethical approval for this study (Approval No. 1806-071-950) was provided by the Institutional Review Board (IRB) of Seoul National University Hospital on 20 June 2018. Written informed consent was waived by the IRB due to the retrospective nature of the study. The manuscript conforms to the Declaration of Helsinki and the STrengthening the Reporting of OBservational studies in Epidemiology statement.

### 2.2. Study Population

After approval from the IRB, we reviewed the electronic medical records of all patients who had undergone cardiac operations at our tertiary university hospital from January 2014 to January 2016. We excluded patients based on the following criteria: when preoperative chest computed tomography (CT) scan was not performed or when CT scan did not include RIJ, redo cardiac surgery in the same patient during the study period, insufficient medical record data, and CVC not required or impossible due to indwelling catheter in RIJ at the operating theatre entrance.

### 2.3. CVC and Data Collection

As a routine institutional protocol, CVC was performed at the RIJ under ultrasonography guidance after anesthesia induction in every patient. All catheters were placed by trainee anesthesiology fellows or senior residents under attending supervision. The type of catheter was determined according to the type of surgery. If RIJ catheterization was unsuccessful, the catheter was instead placed in LIJ or femoral vein.

We reviewed the electronic medical records and recorded the following variables: demographic data, past medical history, American Society of Anesthesiologists physical status, surgery profile, preoperative medication, preoperative laboratory data, and preoperative coagulation profile. Hemodialysis included all types of vascular access, including arteriovenous fistula, arteriovenous graft, and venous catheter. The following data regarding postoperative clinical outcomes were also obtained: continuous renal replacement therapy, surgical site infection, seizure, stroke, mortality, acute renal injury defined according to the KDIGO (Kidney Disease: Improving Global Outcomes), delirium, atrial fibrillation, and length of hospital stay.

### 2.4. CT Review

Only patients were included who underwent a contrast-enhanced chest CT scans which covered the vertebral body level up to C6 before surgery. Chest CT angiography was performed as an institutional standard before cardiac surgery to evaluate unidentified cardiac pathologies as well as atherosclerosis in aorta, coronary arteries, and bypass conduit candidates in coronary artery bypass grafting (CABG). However, preoperative chest CT could not be performed in some urgent cases. Four different CT scanners were used in this study (LightSpeed Ultra, GE Medical Systems, Waukesha, WI, USA; Brilliance-64, Phillips Medical Systems, Best, Netherlands; Sensation 16, SOMATOM Definition, Siemens Medical Solutions, Forchheim, Germany; Aquilion One, Toshiba, Tokyo, Japan). All CT examinations were performed with the following parameters: 120 kVp, 60 to 90 mAs, pitch of 0.75 to 1.5, and collimation of 0.625 to 1.25 mm. All image data were reconstructed using the medium-sharp reconstruction algorithm with a thickness of 1.3 mm or less. Images were acquired during maximum inspiration and breath-holding. Iodinated contrast medium (iopamidol; iodine content, 300 mg/mL) was administered at a rate of 3 mL/s, followed by 30 mL of 0.9% saline chaser at the same rate.

For analysis, the chest CT images were evaluated by anesthesiologists who were blinded to the patient information. CT images were analyzed with a picture archiving and communication systems (PACS) program (M-view, version 5.4; Infinitt Healthcare, Seoul, Korea). The largest level of the RIJ refers to the point where the cross-sectional area (CSA) of the RIJ is largest between the superior endplates of the vertebral body from C6 and C7, which represents the level where CVC is commonly performed. The smallest level of the RIJ refers to the point where the CSA of the RIJ is smallest between the right jugular angle and the largest level in each patient, and it represents the pathway of the central venous guide wire and the catheter. The CSA at both the largest and smallest levels were calculated automatically by directly drawing a line on regions of interest (RIJ) using a polygon area measurement tool within the PACS program [19] (Figure S1). The diameter, which was defined as the mean value of the anterior-posterior diameter and left–-right diameter, was assessed at both levels, as was the RIJ perimeter. CSA ratio was defined as the ratio of the RIJ CSA at the smallest level to the RIJ CSA at the largest level. Other parameters were also calculated, and were defined as diameter ratio and perimeter ratio. Based on a previous study, we defined lower RIJ stenosis as a CSA ratio less than 25% [20]. The shape of the RIJ was not recorded in this study.

### 2.5. Statistical Analysis

The prespecified primary objective of the present study was to determine the incidence of and the risk factors for lower RIJ stenosis in patients undergoing cardiac surgery. Patients were classified into either the stenotic group or the non-stenotic group based on their RIJ CSA ratio.

Statistical analysis was performed with the use of the R statistical package, version 3.6.3 (R Core Team [2016], www.r-project.org, accessed on 20 May 2020). The normality of the data was tested using the Shapiro–Wilk test. The chi-square test was used to compare categorical variables, and the Mann–Whitney U test was used for continuous variables. Univariable logistic regression was performed to screen for risk factors for lower RIJ stenosis. The following predictor variables were included in the univariable analysis of lower RIJ stenosis: age, female, body mass index, smoking, hypertension, diabetes, dyslipidemia, angina, myocardial infarction, hemodialysis, stroke, previous pacemaker, previous IJV catheterization (side not specified), American Society of Anesthesiologists physical status, aspirin, clopidogrel, angiotensin-converting enzyme inhibitor, angiotensin II receptor blocker, β blocker, calcium channel blocker, diuretics, digoxin, oral hypoglycemic agent, warfarin, heparin before CT scan, statin, hematocrit, creatinine, albumin, glucose, C-reactive protein, platelet, left ventricular ejection fraction, activated partial thromboplastin time, fibrinogen, and prothrombin time. Potential risk factors with *p*-values less than 0.2 were included in the multivariable logistic regression. The presence of multicollinearity was examined before modelling the logistic regressions by calculating the variance inflation factor.

An additional prespecified sensitivity analysis was performed to determine the risk factors for lower RIJ stenosis defined by a CSA ratio of <20%. Univariable and multivariable logistic regression analyses were performed in the same manner.

Propensity score matching was applied to extract matched cases for comparison of CT parameters between hemodialysis and non-hemodialysis patients [21]. We used the nearest neighbor-matching method with 1:2 pairing. Variables used for propensity score matching was age, gender, body mass index, hypertension, diabetes, dyslipidemia, and angina. The caliper was defined as 0.2 standard deviations of the logit-transformed propensity score. The CT parameters were compared in the matched sample.

## 3. Results

**Figure 1:**
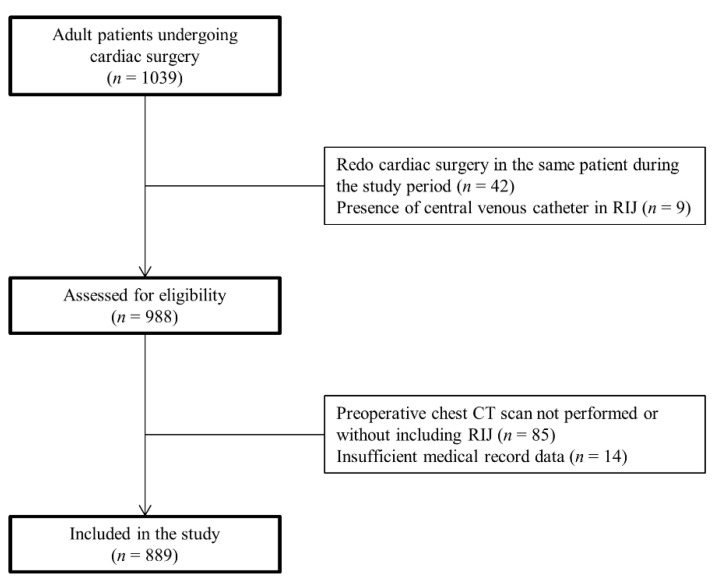
Flow diagram of the patients included in this study. RIJ, right internal jugular vein; CT, computed tomography.

A total of 1039 patients received cardiac surgery during the study period. A total of 150 (14.4%) patients were excluded from the analysis, preoperative chest CT scan not performed or without including RIJ (85 patients), redo cardiac surgery in the same patient during the study period (42 patients), insufficient medical record data (14 patients), or the presence of a central venous catheter in the RIJ at the time of surgical room entrance (nine patients). Finally, a total of 889 patients were enrolled for the data analysis (Figure 1). The types of surgery included CABG only (34.4%), CABG combined with another procedure (12.3%), redo cardiac surgery (8.5%), or other non-CABG operations (44.8%).

**Figure 2:**
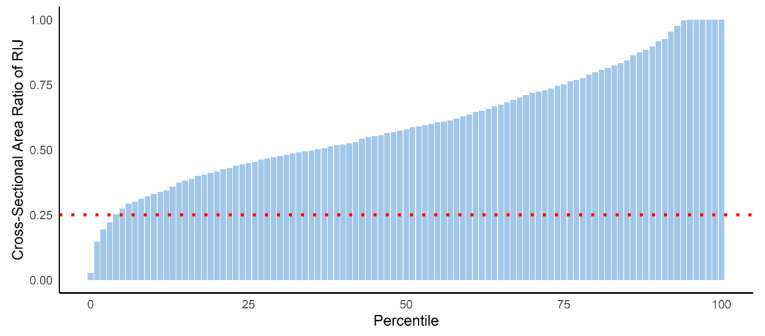
Distribution of the CSA ratio of the RIJ in cardiac surgery patients. The red horizontal line indicates the threshold value for lower RIJ stenosis. CSA, cross-sectional area; RIJ, right internal jugular vein.

Of the 889 patients, 35 (3.9%) had stenosis at the RIJ (stenotic group), and the other 854 (96.1%) patients had a non-stenotic RIJ (non-stenotic group) (Figure 2). There were no significant differences in demographic data in terms of age, sex, or body mass index between groups. However, the stenotic group had a higher proportion of hemodialysis patients than the non-stenotic group (25.7 versus 6.7%, *p* < 0.001) (Table 1). There was no significant difference in the rate of RIJ catheterization between the stenotic group and the non-stenotic group (97.0 versus 97.1%). The intraoperative data and postoperative outcomes did not significantly differ between the two groups (Table 2).

The CT parameters of the two groups are shown in Table 3. Differences in all parameters at the largest level were comparable between groups. Diameter, CSA, and perimeter at the smallest level were all significantly lower in the stenotic group than in the non-stenotic group. The median (interquartile range (IQR)) CSA ratio of the stenotic group was 0.18 (0.14–0.21), compared with 0.59 (0.47–0.76) in the non-stenotic group (*p* < 0.001). The ratio of each parameter at the smallest level to that at the largest level was also significantly lower in the stenotic group than in the non-stenotic group. The ratio of diameter and perimeter was >1 in some patients (36 (4.0%) and 34 (3.8%), respectively), since the definitions of the largest level and the smallest level were based on the CSA.

A univariable logistic regression analysis of the risk factors for lower RIJ stenosis showed that diabetes, dyslipidemia, angina, hemodialysis, previous IJV catheterization, preoperative angiotensin II receptor blocker, and preoperative calcium channel blocker had *p*-values < 0.2. In our multivariable logistic regression analysis, hemodialysis was the only significant independent factor for lower RIJ stenosis (odds ratio (OR), 3.54; 95% confidence interval (CI), 1.472–8.514; *p* = 0.005) (Table 4).

Based on our sensitivity analysis, 21 (2.4%) patients had CSA ratios < 20% (Table S1). In our univariable logistic regression analysis, dyslipidemia, hemodialysis, previous IJV catheterization, preoperative angiotensin II receptor blocker, creatinine, albumin, and platelet count had *p*-values < 0.2. In our multivariable logistic regression analysis, hemodialysis (OR, 10.842; 95% CI, 3.589–32.75; *p* < 0.001) was again the only significant predictor of a CSA ratio < 20%.

In the propensity score matching, the matched sample was comprised of 66 patients in the hemodialysis group and 132 patients in the non-hemodialysis group (Table S2). Two groups were comparable for all covariates with a standardized difference less than 0.20. In the matched cohort, the smallest diameter, CSA, and perimeter in the hemodialysis group were significantly lower than those in the non-hemodialysis group.

## 4. Discussion

This study demonstrates that the incidence of lower RIJ stenosis was up to 3.9% in cardiac surgery patients, and preoperative hemodialysis was significantly associated with an increased risk of lower RIJ stenosis. To the best of our knowledge, this is the first retrospective study to examine the incidence and associated risk factors for lower RIJ stenosis in patients undergoing cardiac surgery. Our study will provide a valuable clinical implications that central venous catheterization in cardiac surgery patients receiving hemodialysis requires a great caution, given that the central venous catheterization is becoming more frequent in cardiac surgery population with the introduction of minimally invasive surgery.

The term lower RIJ stenosis is used clinically as a means of central venous stenosis (CVS) compared with upper RIJ stenosis, which is used in diseases such as multiple sclerosis [22], eagle syndrome [23], and Meniere’s syndrome [24]. Because patients with CVS are often asymptomatic, the actual incidence of CVS is difficult to determine. Previous studies investigated CVS based on CT scans or angiography. These studies revealed that the use of indwelling venous devices such as chemotherapy catheters, cardiac rhythm devices, and peripherally inserted central catheters are risk factors for CVS in patients receiving hemodialysis [11]. In our study, as in previous studies, hemodialysis was an independent risk factor for lower RIJ stenosis; however, the incidence of RIJ stenosis was not very low considering the proportion of patients on hemodialysis undergoing cardiac surgery (7.4%).

In patients on hemodialysis, CVS may occur due to thrombus formation, external compression, or venous wall thickening. Among these factors, venous thrombosis is the most commonly reported reason for lower RIJ stenosis in hemodialysis patients. Thrombus formation can occur within 24 h after catheterization [25], and its incidence increases with the duration of catheter dependence [26]. The most common sites for thrombus formation are the puncture site and the outer surface of the catheter. Apart from the targeted vein access site, adjacent ipsilateral central veins can be narrowed after CVC [27], but stenosis at remote central veins from the venous access site has also been reported [28]. A thrombus can resolve, and this process can be encouraged by removing the central venous catheter, although it may persist for up to several years [29]. In our study, the rate of previously known risk factors of CVS such as previous pacemaker, previous IJV catheterization (side not specified) were not significantly different between the two groups. This might be attributed to the fact that the population of our study were cardiac surgery patients, and not many of them undergo hemodialysis. Considering that the rate of heparin injection was also comparable, it can be inferred that the contribution of thrombus formation in CVS is somewhat limited.

Intraluminal narrowing caused by central venous access is also thought to contribute to the occurrence of CVS. Placement of indwelling intravascular devices can occupy a significant portion of the central veins, which leads to increased blood flow and abnormal shear stress, followed by venous wall hyperplasia and stenosis [11]. This theory is supported by the results of radiologic studies showing that the mean CSA of the LIJ was significantly smaller than that of the RIJ [30,31], making the LIJ more susceptible to catheter-related CVS [32]. This explains the finding in our study that the CSA at the smallest level was lower in hemodialysis patients than in non-hemodialysis patients, but that the CSA at the largest level was comparable between the two patient populations. The creation of arteriovenous fistulae can also markedly increase venous flow in the upper extremities, resulting in turbulence across the anatomically narrow site and venous valves. This leads to platelet deposition and intimal hyperplasia, and finally CVS [33]. This suggests that CVS may also develop in patients on chronic hemodialysis, which is performed via an anatomical shunt not involving the jugular veins.

The normal shape of the IJV is known to be conical [30], consistent with our findings that the smallest level in our CT finding was usually located in the venous angle, which is the lower end of IJV. This means that the CSA of the RIJ in the thorax is considerably smaller than in the neck level but without clinical significance in the normal population. In hemodialysis group, the median CSA at the largest level was relatively similar to that in non-hemodialysis group, whereas the median CSA at the smallest level was significantly smaller. This suggests that ultrasonography scanning of the RIJ before CVC cannot guarantee the patency of the lower RIJ, which mandates the need for a radiologic evaluation in high risk patients for lower RIJ stenosis. However, our results demonstrated that the rate of RIJ catheterization was not significantly different regardless of hemodialysis before surgery. This might be illuminated in several ways. First of all, due to the very high success rate of RIJ catheterization, the sample size of our study may not be sufficient to ensure an adequate power to detect the differences. Second, the occurrence of CVC failures linked with intrathoracic narrowing of RIJ is attributed to the unsuccessful advancement of central venous catheter. Because the outer diameter of the 9Fr catheter is 3 mm, it can be placed intravenously using the Seldinger technique without difficulty once the guiding wire is in advance placed at correct position. However, the minimum vessel diameter does not always ensure catheterization success, and vascular stenosis always has the potential to increase the risk of catheterization failure. Nevertheless, due to the retrospective design of the study, it was not possible to confirm whether LIJ catheterization was due to RIJ catheterization failure or other reasons, which requires further prospective study.

The current study evaluated the RIJ using contrast-enhanced chest CT covering the vertebral body level up to C6. As this was part of the routine preoperative evaluation at our institution for patients undergoing cardiac surgery, no extra radiation exposure to the patients was necessary for internal jugular vein assessment. If preoperative chest CT in cardiac surgery patients shows that the lower RIJ is stenotic, anesthesiologists should choose an alternative site to prevent unnecessary cannulation or complications.

The present study had several limitations. First, it is a retrospective analysis, which is subject to bias from unmeasured factors. Although we included the history of previous IJV catheterization in the analysis as covariates, we were unable to identify the number of instances of previous venous catheter access, which is a known risk factor for CVS in hemodialysis patients. Hemodialysis patients usually have a history of multiple hemodialysis catheter insertions. Patients undergoing heart surgery often have several comorbidities, and therefore are likely to have a history of central venous catheter access. Further, as our study was exploratory, our results do not infer causality and the associations should be interpreted with caution. Second, we focused on CT images of the CSA at levels from the superior endplates of the C6 vertebral body to the right jugular angle, where the RIJ was perpendicular to the transverse plane. Additional studies are needed to evaluate the lower pathway of the RIJ catheter, which includes the right innominate vein and superior vena cava in cardiac surgery patients, as they can also contribute to RIJ catheterization failure. Finally, since CT images were taken in awake, non-intubated, spontaneously breathing patients in the supine position, the CSA of the RIJ may have been underestimated.

## 5. Conclusions

In conclusion, the incidence of lower RIJ stenosis on preoperative CT scans with contrast was 3.9% in cardiac surgery patients. Preoperative hemodialysis was significantly associated with an increased risk of lower RIJ stenosis in patients undergoing cardiac surgery. Extra care is needed during central venous catheterization in hemodialysis patients undergoing cardiac surgery.

## Figures and Tables

**Table 1:** Baseline characteristics of patients undergoing cardiac surgery with or without lower RIJ stenosis.

	Stenotic Group	Non-Stenotic Group	*p*-Value
	(*n* = 35)	(*n* = 854)	
*Demographics*			
Age (years)	63 (56.5–70.5)	64.5 (55–72)	0.935
Female	11 (31.4%)	322 (37.7%)	0.566
Body mass index (kg/m^2^)	23.1 (21.7–26.0)	23.8 (21.5–26.0)	0.989
Smoking	8 (22.9%)	157 (18.4%)	0.656
*Past medical history*			
Hypertension	18 (51.4%)	411 (48.1%)	0.833
Diabetes	14 (40.0%)	221 (25.9%)	0.097
Dyslipidemia	9 (25.7%)	147 (17.2%)	0.285
Angina	19 (54.3%)	330 (38.6%)	0.093
Myocardial infarction	3 (8.6%)	58 (6.8%)	0.946
Hemodialysis	9 (25.7%)	57 (6.7%)	<0.001
Stroke	4 (11.4%)	132 (15.5%)	0.682
Previous pacemaker	1 (2.9%)	18 (2.1%)	1.000
Previous IJV catheterization (side not specified)	5 (14.3%)	54 (6.3%)	0.131
ASA physical status			0.896
1	0 (0.0%)	4 (0.5%)	
2	7 (20.0%)	224 (26.2%)	
3	27 (77.1%)	592 (69.3%)	
4	1 (2.9%)	33 (3.9%)	
5	0 (0.0%)	1 (0.1%)	
*Surgery profiles*			
CABG only	17 (48.6%)	289 (33.8%)	0.106
Combined CABG and valvular	1 (2.9%)	108 (12.6%)	0.142
Cardiac redo before the study period	3 (8.6%)	73 (8.5%)	1.000
Emergency	5 (14.3%)	116 (13.6%)	1.000
*Preoperative medication*			
Aspirin	14 (40.0%)	290 (34.0%)	0.578
Clopidogrel	7 (20.0%)	139 (16.3%)	0.726
ACE inhibitor	2 (5.7%)	69 (8.1%)	0.851
ARB	14 (40.0%)	247 (28.9%)	0.222
β blocker	6 (17.1%)	142 (16.6%)	1.000
Calcium channel blocker	17 (48.6%)	317 (37.1%)	0.233
Diuretics	14 (40.0%)	288 (33.7%)	0.558
Digoxin	5 (14.3%)	92 (10.8%)	0.706
Oral hypoglycemic agent	8 (22.9%)	182 (21.3%)	0.993
Warfarin	5 (14.3%)	130 (15.2%)	1.000
Heparin before CT scan	3 (8.6%)	72 (8.4%)	1.000
Statin	12 (34.3%)	300 (35.1%)	1.000
*Preoperative laboratory data*			
Hematocrit (%)	35.4 (33.6–38.5)	34.7 (32.0–37.5)	0.250
Creatinine (mg/dL)	1.0 (0.8–1.3)	0.9 (0.8–1.1)	0.492
Albumin (g/dL)	4.1 (3.6–4.4)	4.1 (3.7–4.3)	0.904
Glucose (mg/dL)	140 (125.5–169.5)	131 (102–168)	0.113
C-reactive protein (mg/L)	0.2 (0.1–0.6)	0.2 (0.1–0.6)	0.835
Platelet (×10^9^/L)	223 (176–262)	204 (167–249)	0.394
LV ejection fraction (%)	56 (53–63)	58 (53–63)	0.754
*Preoperative coagulation profile*			
aPTT (s)	33.4 (31.0–39.2)	32.8 (30.6–36.9)	0.924
Fibrinogen (mg/dL)	322 (266.5–362.5)	309 (262–370)	0.588
Prothrombin time (INR)	1.0 (1.0–1.1)	1.0 (1.0–1.1)	0.923

Values are expressed as mean ± SD, median (IQR) or number (%). RIJ, right internal jugular vein; IJV, internal jugular vein; ASA, American Society of Anesthesiologists; CABG, coronary artery bypass grafting; ACE, angiotensin-converting enzyme; ARB, angiotensin II receptor blocker; CT, computed tomography; LV, left ventricular; aPTT, activated partial thromboplastin time.

**Table 2:** Intraoperative data and postoperative outcomes of patients undergoing cardiac surgery with or without lower RIJ stenosis.

	Stenotic Group	Non-Stenotic Group	*p*-Value
	(*n* = 35)	(*n* = 854)	
Duration of surgery (min)	370 (315–441)	380 (325–455)	0.741
Duration of anesthesia (min)	455 (395–515)	455 (396–534)	0.803
CRRT	2 (5.7%)	19 (2.2%)	0.445
Surgical site infection	0 (0.0%)	15 (1.8%)	0.903
Seizure	0 (0.0%)	9 (1.1%)	1.000
Stroke	2 (5.7%)	30 (3.5%)	0.824
Death from any cause	3 (8.6%)	62 (7.3%)	1.000
Death from cardiac cause	1 (2.9%)	30 (3.5%)	1.000
In-hospital mortality	1 (2.9%)	22 (2.6%)	1.000
KDIGO stage			0.465
0	19 (54.3%)	512 (60.0%)	
1	8 (22.9%)	218 (25.5%)	
2	3 (8.6%)	62 (7.3%)	
3	5 (14.3%)	62 (7.3%)	
Delirium	5 (14.3%)	103 (12.1%)	0.896
Atrial fibrillation	6 (17.1%)	183 (21.4%)	0.692
Postoperative length of hospital stay (days)	18 (14.5–23)	17 (13–25)	0.339

Values are expressed as median (IQR) or number (%). RIJ, right internal jugular vein; CRRT, continuous renal replacement therapy; KDIGO, kidney disease: improving global outcomes.

**Table 3:** Comparison of CT parameters of RIJ between stenotic group and non-stenotic group.

	Stenotic Group	Non-Stenotic Group	*p*-Value
	(*n* = 35)	(*n* = 854)	
Smallest diameter (mm)	5 (3.5–7.2)	10.6 (8.5–12.6)	<0.001
Smallest CSA (mm^2^)	27.2 (13.7–37.9)	88.5 (61.8–119.6)	<0.001
Smallest perimeter (mm)	20.3 (13.5–24.6)	32.6 (27.1–37.9)	<0.001
Largest diameter (mm)	13.4 (11.3–16.8)	14.1 (11.3–16.8)	0.791
Largest CSA (mm^2^)	143.7 (89.9–192.7)	153.6 (104.1–209.6)	0.496
Largest perimeter (mm)	41.6 (33.2–51.0)	43.0 (35.2–50.6)	0.743
Diameter ratio	0.37 (0.34–0.47)	0.76 (0.66–0.88)	<0.001
CSA ratio	0.18 (0.14–0.21)	0.59 (0.47–0.76)	<0.001
Perimeter ratio	0.47 (0.40–0.50)	0.77 (0.67–0.88)	<0.001

Values are expressed as median (IQR). RIJ, right internal jugular vein; CSA, cross-sectional area.

**Table 4:** Univariable and multivariable logistic regression analysis of lower RIJ stenosis in patients undergoing cardiac surgery.

	Univariable Regression Analysis		Multivariable Regression Analysis	
	Unadjusted OR (95% CI)	*p*-Value	Adjusted OR (95% CI)	*p*-Value
Age (years)	1.001 (0.976–1.027)	0.939		
Female	0.757 (0.366–1.567)	0.453		
Body mass index (kg/m^2^)	1.036 (0.939–1.142)	0.481		
Smoking	1.315 (0.586–2.95)	0.506		
Hypertension	1.141 (0.58–2.244)	0.702		
Diabetes	1.91 (0.955–3.82)	0.068	1.36 (0.639–2.891)	0.425
Dyslipidemia	1.665 (0.764–3.627)	0.199	1.235 (0.527–2.894)	0.627
Angina	1.886 (0.956–3.719)	0.067	1.233 (0.564–2.698)	0.600
Myocardial infarction	1.287 (0.382–4.328)	0.684		
Hemodialysis	4.84 (2.166–10.817)	<0.001	3.54 (1.472–8.514)	0.005
Stroke	0.706 (0.245–2.032)	0.518		
Previous pacemaker	1.366 (0.177–10.533)	0.765		
Previous IJV catheterization (side not specified)	2.469 (0.921–6.619)	0.072	1.666 (0.574–4.838)	0.348
ASA physical status	1.253 (0.644–2.437)	0.507		
Aspirin	1.297 (0.65–2.587)	0.461		
Clopidogrel	1.286 (0.551–3.002)	0.561		
ACE inhibitor	0.69 (0.162–2.935)	0.615		
ARB	1.638 (0.82–3.274)	0.162	1.402 (0.682–2.883)	0.358
β blocker	1.037 (0.423–2.545)	0.936		
Calcium channel blocker	1.6 (0.813–3.149)	0.174	1.259 (0.617–2.568)	0.527
Diuretics	1.31 (0.657–2.615)	0.444		
Digoxin	1.38 (0.523–3.646)	0.515		
Oral hypoglycemic agent	1.094 (0.489–2.449)	0.827		
Warfarin	0.928 (0.354–2.436)	0.88		
Heparin before CT scan	1.018 (0.304–3.407)	0.977		
Statin	0.963 (0.473–1.964)	0.918		
Hematocrit (%)	1.048 (0.969–1.134)	0.241		
Creatinine (mg/dL)	1.099 (0.912–1.324)	0.322		
Albumin (g/dL)	0.761 (0.393–1.475)	0.419		
Glucose (mg/dL)	1.002 (0.996–1.008)	0.571		
C-reactive protein (mg/L)	1.002 (0.905–1.109)	0.971		
Platelet (×10^9^/L)	1 (0.995–1.005)	0.949		
LV ejection fraction (%)	1.001 (0.971–1.032)	0.946		
aPTT (s)	0.999 (0.981–1.019)	0.952		
Fibrinogen (mg/dL)	1.001 (0.997–1.005)	0.753		
Prothrombin time (INR)	0.549 (0.068–4.456)	0.575		

RIJ, right internal jugular vein; IJV, internal jugular vein; ASA, American Society of Anesthesiologists; ACE, angiotensin-converting enzyme; ARB, angiotensin II receptor blocker; CT, computed tomography; LV, left ventricular; aPTT, activated partial thromboplastin time.

## Data Availability

Not applicable.

## Supplementary Materials

The following are available online at https://www.mdpi.com/2077-0383/10/5/1042/s1, Table S1: Univariable and multivariable logistic regression analysis of CSA ratio < 20% in patients undergoing cardiac surgery, Table S2: Comparison of CT parameters of RIJ between hemodialysis group and non-hemodialysis group in matched patients, Figure S1: A CT scan demonstrating the measurement of a cross-sectional area of the right internal jugular vein using a polygon area measurement tool.
