# Supplementary material for: Hemodialysis as a Risk Factor for Lower Right Internal Jugular Stenosis in Cardiac Surgery Patients: A Retrospective Single-Center Study
Source: J Clin Med. 2021 Mar 3;10(5):1042. doi: 10.3390/jcm10051042 (PMC7959313; doi:10.3390/jcm10051042)
Supplement: Supplementary file 1 [file jcm-10-01042-s001.zip › Table S1_JCM.docx]

**Table S1.** Univariable and multivariable logistic regression analysis of CSA ratio <20% in patients undergoing cardiac surgery

|  | Univariable regression analysis | | Multivariable regression analysis | |
| --- | --- | --- | --- | --- |
|  | Unadjusted OR (95% CI) | P-value | Adjusted OR (95% CI) | P-value |
| Age (years) | 0.989(0.959-1.021) | 0.496 |  |  |
| Female | 0.662(0.254-1.722) | 0.398 |  |  |
| Body mass index (kg/m^2^) | 1.008(0.888-1.144) | 0.9 |  |  |
| Smoking | 1.383(0.499-3.83) | 0.533 |  |  |
| Hypertension | 1.184(0.498-2.817) | 0.702 |  |  |
| Diabetes | 1.738(0.711-4.247) | 0.226 |  |  |
| Dyslipidemia | 1.915(0.731-5.015) | 0.186 | 1.311(0.456-3.768) | 0.615 |
| Angina | 1.725(0.725-4.105) | 0.218 |  |  |
| Myocardial infarction | 1.443(0.328-6.346) | 0.627 |  |  |
| Hemodialysis | 8.594(3.424-21.568) | <0.001 | 10.842(3.589-32.75) | <0.001 |
| Stroke | 0.577(0.133-2.503) | 0.462 |  |  |
| Previous pacemaker | 2.361(0.3-18.561) | 0.414 |  |  |
| Previous IJV catheterization  (side not specified) | 3.478(1.132-10.691) | 0.03 | 2.536(0.735-8.751) | 0.141 |
| ASA physical status | 1.399(0.592-3.305) | 0.444 |  |  |
| Aspirin | 1.457(0.607-3.497) | 0.4 |  |  |
| Clopidogrel | 0.845(0.246-2.906) | 0.789 |  |  |
| ACE inhibitor | 0(0-Inf) | 0.99 |  |  |
| ARB | 1.833(0.763-4.405) | 0.175 | 1.995(0.796-4.998) | 0.14 |
| β blocker | 1.584(0.571-4.394) | 0.377 |  |  |
| Calcium channel blocker | 1.526(0.641-3.634) | 0.339 |  |  |
| Diuretics | 1.472(0.613-3.533) | 0.387 |  |  |
| Digoxin | 1.961(0.646-5.951) | 0.235 |  |  |
| Oral hypoglycemic agent | 1.487(0.569-3.886) | 0.418 |  |  |
| Warfarin | 1.324(0.438-3.996) | 0.619 |  |  |
| Heparin before CT scan | 0.536(0.071-4.054) | 0.546 |  |  |
| Statin | 0.923(0.369-2.311) | 0.864 |  |  |
| Hematocrit (%) | 0.984(0.888-1.091) | 0.76 |  |  |
| Creatinine (mg/dL) | 1.165(0.956-1.418) | 0.129 | 0.798(0.609-1.045) | 0.101 |
| Albumin (g/dL) | 0.587(0.263-1.309) | 0.193 | 0.695(0.287-1.684) | 0.42 |
| Glucose (mg/dL) | 1.001(0.993-1.01) | 0.725 |  |  |
| C-reactive protein (mg/L) | 1.03(0.931-1.139) | 0.565 |  |  |
| Platelet (×10^9^/L) | 0.995(0.988-1.002) | 0.126 | 0.995(0.988-1.002) | 0.181 |
| LV ejection fraction (%) | 1.013(0.971-1.057) | 0.547 |  |  |
| aPTT (sec) | 0.998(0.972-1.025) | 0.894 |  |  |
| Fibrinogen (mg/dL) | 0.998(0.992-1.003) | 0.431 |  |  |
| Prothrombin time (INR) | 0.317(0.016-6.367) | 0.453 |  |  |

RIJ, right internal jugular vein; IJV, internal jugular vein; ASA, American Society of Anesthesiologists; ACE, angiotensin-converting enzyme; ARB, angiotensin II receptor blocker; CT, computed tomography; LV, left ventricular; aPTT, activated partial thromboplastin time.
