# Supplementary material for: Hemodialysis as a Risk Factor for Lower Right Internal Jugular Stenosis in Cardiac Surgery Patients: A Retrospective Single-Center Study
Source: J Clin Med. 2021 Mar 3;10(5):1042. doi: 10.3390/jcm10051042 (PMC7959313; doi:10.3390/jcm10051042)
Supplement: Supplementary file 1 [file jcm-10-01042-s001.zip › Table S2_JCM.docx]

**Supplementary Table 2.** Comparison of CT parameters of RIJ between hemodialysis group and non-hemodialysis group in matched patients

|  | Hemodialysis group | Non-hemodialysis group | P-value |
| --- | --- | --- | --- |
|  | (n=66) | (n=132) |  |
| Smallest diameter (mm) | 9.60 (6.99-11.25) | 10.41 (8.10-12.43) | 0.024 |
| Smallest CSA (mm^2^) | 65.25 (40.58-99.81) | 83.25 (56.95-113.20) | 0.018 |
| Smallest perimeter (mm) | 28.33 (22.13-34.67) | 31.63 (26.41-36.70) | 0.019 |
| Largest diameter (mm) | 12.80 (10.89-15.46) | 13.47 (11.61-17.13) | 0.19 |
| Largest CSA (mm^2^) | 126.41 (89.95-172.68) | 148.24 (102.70-217.55) | 0.129 |
| Largest perimeter (mm) | 39.48 (32.53-46.18) | 41.99 (34.33-51.08) | 0.189 |
| Diameter ratio | 0.69 (0.61-0.86) | 0.75 (0.66-0.86) | 0.126 |
| CSA ratio | 0.49 (0.40-0.81) | 0.57 (0.43-0.75) | 0.178 |
| Perimeter ratio | 0.75 (0.61-0.86) | 0.78 (0.67-0.88) | 0.089 |

Values are expressed as median (IQR). The following covariates were used for matching: age, sex, body mass index, diabetes, dyslipidemia, hypertension, and angina.

CT, computed tomography; RIJ, right internal jugular vein; CSA, cross-sectional area.
