# Supplementary figures and images for: Hemodialysis as a Risk Factor for Lower Right Internal Jugular Stenosis in Cardiac Surgery Patients: A Retrospective Single-Center Study
Source: J Clin Med. 2021 Mar 3;10(5):1042. doi: 10.3390/jcm10051042 (PMC7959313; doi:10.3390/jcm10051042)

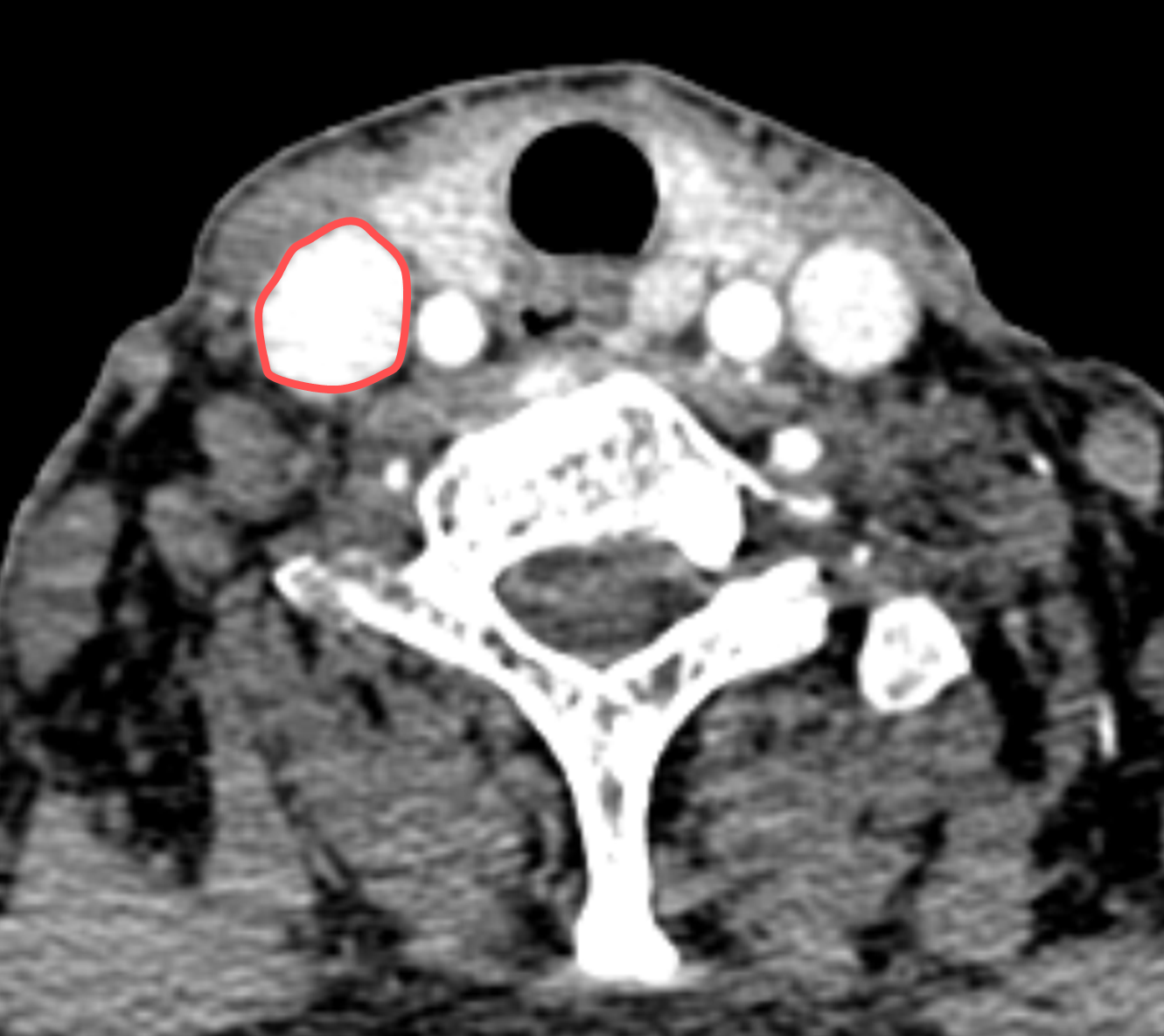

Supplement: Supplementary file 1 [file jcm-10-01042-s001.zip › Figure S1.tif]
